# Severe acute respiratory coronavirus virus 2 (SARS-CoV-2) RNA and viable virus contamination of hospital emergency department surfaces and association with patient coronavirus disease 2019 (COVID-19) status and aerosol-generating procedures

**DOI:** 10.1017/ice.2023.183

**Published:** 2024-02

**Authors:** Scott C. Roberts, Elliana S. Barbell, Douglas Barber, Suzanne E. Dahlberg, Robert Heimer, Karen Jubanyik, Vivek Parwani, Melinda M. Pettigrew, Jason M. Tanner, Andrew Ulrich, Martina Wade, Anne L. Wyllie, Devyn Yolda-Carr, Richard A. Martinello, Windy D. Tanner

**Affiliations:** 1 Department of Internal Medicine, Yale University, New Haven, Connecticut; 2 Department of Epidemiology of Microbial Diseases, Yale School of Public Health, New Haven, Connecticut; 3 Infection Prevention, Yale New Haven Hospital, New Haven, Connecticut; 4 Department of Emergency Medicine, Yale University, New Haven, Connecticut

## Abstract

Emergency departments are high-risk settings for severe acute respiratory coronavirus virus 2 (SARS-CoV-2) surface contamination. Environmental surface samples were obtained in rooms with patients suspected of having COVID-19 who did or did not undergo aerosol-generating procedures (AGPs). SARS-CoV-2 RNA surface contamination was most frequent in rooms occupied by coronavirus disease 2019 (COVID-19) patients who received no AGPs.

The emergency department (ED) serves as the gateway for hospital admission for severe coronavirus disease 2019 (COVID-19). Many of these patients require respiratory aid via aerosol-generating procedures (AGPs), which can contaminate environmental surfaces through aerosol deposition. Severe acute respiratory coronavirus virus 2 (SARS-CoV-2) environmental contamination after procedures carries implications for transmission, and characterizing this contamination can guide infection prevention practices. We determined the occurrence and viability of SARS-CoV-2 on ED surfaces when COVID-19 patients did and did not receive AGPs.

## Methods

Patients presenting to the Yale New Haven Hospital Adult ED, composed of 101 beds across 2 campuses, were evaluated for COVID-19. From January through December 2021, environmental swabs were collected from a convenience sample of rooms housing patients actively infected with or under investigation for COVID-19. Sampling was prioritized for rooms of patients receiving AGPs. AGPs were classified as endotracheal intubation or extubation, manual bag-valve-mask (BVM) ventilation, cardiopulmonary resuscitation, noninvasive positive-pressure ventilation (NPPV), high-flow oxygenation, bronchoscopy, and nebulizer therapy.^1^

From each room, 5 samples were collected while occupied by the patient or immediately following discharge or transfer, but prior to cleaning. Among these samples, 4 were from fixed surfaces, selected based on touch frequency and aerosol source proximity: high-touch surfaces within and farther than 2 m (6 feet) from the patient (bedrail, door handle, respectively), and low-touch surfaces within and farther than 2 m (6 feet) from the patient: vital signs monitor frame, air return vent (standard rooms) or procedure light (resuscitation rooms), respectively. A fifth sample was taken from the reusable AGP equipment or oxygen gauge behind the bed. Surface swabs underwent RNA extraction and RT-qPCR using N1 primers, and RNA copies were quantified via a standard curve using controls of known copy number. Positive samples were cultured using Vero E6 cells and were examined for cytopathic effect. Severity was classified using a previously validated COVID-19 ordinal severity index.^2^ Differences between AGP and non-AGP room SARS-CoV-2 RNA contamination frequency were analyzed with the Fisher exact test and concentrations were analyzed with the Kruskal-Wallis test. Additional methods are in the Supplementary Material (online).

## Results

Sample collection yielded 1,010 environmental specimens from 202 rooms. Room types included resuscitation bays (n = 69, 34.2%), airborne-infection isolation rooms (n = 56, 27.7%), and standard rooms (n = 77, 38.1%). AGPs were performed in 157 rooms (77.7%), and included intubation (n = 52), NPPV (n = 47 BiPAP and 2 CPAP), high-flow oxygenation (n = 34), nebulizer therapy (n = 13), and BVM ventilation (n = 1), or multiple AGPs (n = 8). Of 202 rooms, 87 housed SARS-CoV-2–positive patients (43.1%), approximately half of whom received AGPs (n = 42, 48.3%). These included high-flow oxygenation (n = 28), NPPV (n = 4), nebulizers (n = 4), intubation (n = 4), or multiple (n = 2).

In total, 36 swabs (3.6%, n = 1,010) from 29 rooms (14.4%, n = 202) were positive for SARS-CoV-2 RNA (Table 1). Of 87 COVID-19 patient rooms, 19 (21.8%) had SARS-CoV-2 RNA on at least 1 surface, including 13 rooms (28.9%, n = 45) where AGPs were not performed and 6 rooms (14.3%, n = 42) where AGPs were performed (*P* = .123). Patients spent more time in the room prior to environmental swab collection when surfaces were SARS-CoV-2 RNA-positive (mean, 295.4 minutes; n = 19) versus negative (mean, 223.8 minutes; n = 68), but this was not statistically significant (*P* = .213). SARS-CoV-2 RNA was detected in 10 rooms (8.7%, n = 115) occupied by SARS-CoV-2–negative patients. The mean estimated concentration of SARS-CoV-2 RNA on contaminated surfaces was 24.1 (median, 20.2) copies per 100 cm^2^ in rooms housing SARS-CoV-2–negative patients, 52.1 (median, 48.1) copies per 100 cm^2^ in rooms housing COVID-19 patients who underwent AGPs, and 147.6 (median, 43.7) copies per 100 cm^2^ in rooms housing COVID-19 patients who did not undergo an AGP (*P* = .239).

**Table 1.** Percentage of Rooms and Surface Swabs Testing Positive for SARS-CoV-2 RNA or Infectious Virus by RT-qPCR or Culture

COVID-19 Status	Aerosol-Generating Procedure (AGP)	Rooms Sampled, No.	Rooms (%) Positive for SARS-CoV-2 RNA, No. (%)	Swabs Collected, No.	Swabs Positive for SARS-CoV-2 RNA, No. (%)	Average SARS-CoV-2 RNA Copies per 100 cm^2^ Swabbed Surface Area^a^	Swabs Positive by Viral Tissue Culture, No.
Positive	Yes	42	6 (14.3)	210	8 (3.8)	52.1	0
Positive	No	45	13 (28.9)	225	16 (7.1)	147.6	1
Negative	Yes	115	10 (8.7)	575	12 (2.1)	24.1	0
Total		202	29 (14.4)	1,010	36 (3.6)		**1**

Note. RT-qPCR, reverse-transcription quantitative polymerase chain reaction.

a Mean copies, as determined by RT-qPCR curve determined from known quantities of PCR.

Of the 6 SARS-CoV-2 RNA-contaminated rooms of SARS-CoV-2–positive patients where AGPs occurred, high-flow oxygenation occurred in 5 rooms (n = 28), and nebulizer therapy occurred in 1 room (n = 4). No SARS-CoV-2–positive patient room surfaces were contaminated after intubation (n = 4) or NPPV (n = 4).

SARS-CoV-2 RNA was most frequently detected on air-duct return vents (13 of 133, 9.8%), followed by bedrails (10 of 202, 5.0%), reusable equipment (4 of 202, 2.0%), monitors (4 of 202, 2.0%), door handles (3 of 202, 1.5%), and procedure lights (2 of 69, 2.9%) (Table 2). SARS-CoV-2 RNA contamination ranged from 5–687 copies per 100 cm^2^ on vents, 6–74 copies per 100 cm^2^ on bedrails, 32–39 copies per 100 cm^2^ on door handles, 16–65 copies/100 cm^2^ on reusable equipment, and 22–504 copies per 100 cm^2^ on the monitor frame (Table 2 and Supplementary Table 1 online). Most contaminated rooms had only 1 SARS-CoV-2–positive surface; however, 6 rooms had multiple positive surfaces. A higher percentage of nonresuscitation rooms were positive (17.9%, n = 24 of 134) than resuscitation rooms (7.4%, n = 5 of 68; *P* = .055).

**Table 2.** Emergency Department Room Surfaces Positive for SARS-CoV-2 Contamination

				RNA Copies/cm^2^			
Surface	Surface Area Sampled (cm^2^)	Samples Collected, No.	Positive Surface Swabs, No. (%)	Total	Mean	Range	IQR
High touch, near patient (< 2 m): bedrails	129	202	10 (5.0)	84.8	15.9	4.7–538.1	8.7–70.2
High touch, distant from patient (>2 m): door handle	65	202	3 (1.5)	35.9	36.1	32.3–39.4	34.2–37.7
Low touch, distant from patient (>2 m):							
Air return vent Resuscitation room procedure lights	15 232	133 69	13 (9.8) 2 (2.9)	91.1 31.0	28.6 31.0	5.0–686.6 11.9–50.1	9.6–71.7 21.4–40.5
Low touch, near patient (<2 m): vital signs monitor frame	194	202	4 (2.0)	178.0	92.5	22.2–504.7	33.4–237.1
**Reusable AGP equipment**							
Glidescope	155	4	0 (0.0)	…			
Noninvasive positive pressure ventilation control screen frame (BiPAP; CPAP)	226	53	0 (0.0)	…			
High-flow oxygen control	219	36	1 (2.8)	15.9	15.9	…	…
Oxygen gauge (swabbed for nebulizer treatments and when no AGP‡ was administered)	97	56	1 (1.8)	64.8	64.8	…	…
Mechanical ventilation control screen frame	226	49	2 (4.1)	36.3	36.3	25.8–46.8	31.0–41.6

Note. BiPAP, bilevel positive airway pressure; CPAP, continuous positive airway pressure; AGP, aerosol-generating procedure.

Of the 36 SARS-CoV-2 RNA-positive samples (bedrail, non-AGP COVID-19 patient), 1 sample was positive by viral tissue culture, exhibiting extensive cytopathic effect. SARS-CoV-2 RNA copy number in pre- and post-incubation tissue culture medium went from undetectable to 3.5 × 10^8^ copies, respectively.

COVID-19 patients occupied 13 of the 202 sampled rooms (6.4%) immediately preceding the occupant present during sampling. One of these rooms had an equipment surface positive for SARS-CoV-2 RNA while occupied by the subsequent SARS-CoV-2–negative patient.

The median severity of patient illness on arrival was 5.0 (IQR, 2.5–6.0) in rooms with SARS-CoV-2 contamination compared to 6.0 (IQR, 4.75–6.0) in rooms without contamination (*P* = .259). The median number of days from symptom onset to ED presentation was 4.0 (IQR, 3.0–7.0) in rooms with detectable SARS-CoV-2 contamination compared to 7.0 (IQR, 3.0–8.5) in rooms without (*P* = .507) (Supplementary Fig. 1A and B online).

## Discussion

SARS-CoV-2 RNA contamination was detected on at least 1 surface in >20% of rooms housing patients with COVID-19. Surface contamination was detected more frequently in rooms of COVID-19 patients who did not have an AGP. We suspect that this finding is due to the natural progression of COVID-19 in which viral loads peak around symptom onset, and more severe disease occurs later in the hyperinflammatory phase of illness when viral load is diminished.^3,4^ This carries infection control implications; mitigating transmission earlier in the disease course when viral transmission potential is greatest may be more impactful, regardless of aerosol deposition.

We observed SARS-CoV-2 contamination in 9% of SARS-CoV-2–negative patient rooms. Given the rapid turnover of ED rooms, prior patients could have contributed to contamination, especially on air-return vents because these are not disinfected between patients. This finding also highlights the ability of upward airflow, even in rooms maintained without negative pressure, to move SARS-CoV-2 aerosols to surfaces unlikely to be implicated in viral transmission.

One sample grew SARS-CoV-2 in tissue culture. Infectious virus has rarely been recovered from hospital surfaces, and positive surfaces are typically within close range of the patient.^5–7^ SARS-CoV-2 remains viable on surfaces up to 21 days, with a half-life of ∼2–5 days,^8^ whereas SARS-CoV-2 RNA exhibits a 1-log reduction over the same period.^9^ Therefore, failure to detect viable virus in more samples with high viral concentrations is not unexpected and supports the evidence of a minimal role of surface and fomite transmission in SARS-CoV-2 spread.^10^ Study limitations include observations at a single emergency department and limited comparisons between AGPs due to small sample sizes. It is unclear whether these findings would be replicated in asymptomatic COVID-19 populations.

## Supplementary material

For supplementary material accompanying this paper visit https://doi.org/10.1017/ice.2023.183. click here to view supplementary material
