# Supplementary material for: Severe acute respiratory coronavirus virus 2 (SARS-CoV-2) RNA and viable virus contamination of hospital emergency department surfaces and association with patient coronavirus disease 2019 (COVID-19) status and aerosol-generating procedures
Source: Infect Control Hosp Epidemiol. 2023 Sep 28;45(2):244–6. doi: 10.1017/ice.2023.183 (PMC10877528; doi:10.1017/ice.2023.183)

Supplemental appendix

*COVID-19 testing*

Active SARS-CoV-2 infection was defined as a new positive SARS-CoV-2 test within the 10 days preceding emergency department (ED) presentation, without a prior positive test in the preceding 90 days. COVID-19 status at the time of the ED encounter through 14 days was verified using the electronic medical record. Nucleic acid amplification testing for SARS-CoV-2 in ED patients was performed on nasopharyngeal or mid-turbinate nasal specimens using U.S. Food and Drug Administration emergency use authorized platforms.

*Cleaning and disinfection protocols*

Accessible surfaces and non-disposable medical equipment were cleaned and disinfected using a disinfectant effective against SARS-CoV-2 prior to the next occupant. Environmental samples collected from rooms where patients died or were discharged prior to clinical SARS-CoV-2 testing were excluded from the analysis. The study protocol was approved by the Yale University Institutional Review Board (IRB) and the Yale Human Research Protection Program (IRB# 2000029221).

*SARS-CoV-2 RNA and infectious SARS-CoV-2 detection*

Environmental swabs were taken from a convenience sample of patients during the week in daytime hours. Samples were collected using SANI-MacroSwabs (Sanigen Co., Ltd, Anyang-si, Gyeonggi-do, South Korea) saturated with a viral transport medium containing no agents known to deactivate SARS-CoV-2. Environmental swab elution and viral deactivation were conducted under biosafety-level 2+ conditions. Tissue culture work was conducted in the biosafety-level 3 laboratory facilities at Yale University. All laboratory protocols were approved by the Yale University Biological Safety Committee. Samples were stored at 4°C up to 72 hours or frozen at -80°C until processing. Swabs were eluted with 5 mL sterile phosphate buffered saline (PBS) and pulse vortexed for 2 minutes. One milliliter of swab eluate was aliquoted for RNA extraction using the Qiagen RNeasy Mini® RNA extraction kit (Qiagen, Hilden Germany). The remaining 4 mL eluate was frozen at -80°C for tissue culture of samples positive for SARS-CoV-2 RNA by reverse transcriptase quantitative PCR (RT-qPCR). RT-qPCR testing of environmental samples utilized the U.S. Centers for Disease Control (CDC) N1 primer/probe set.^1^ The CDC human RNase P (RP) extraction control primers were not utilized because the samples were not directly from humans. RNA copies for environmental samples were determined by generating a standard curve for each RT-qPCR run utilizing C_t_ values and known copy numbers of a SARS-CoV-2 RNA synthetic control (Twist Bioscience, San Francisco USA) at 10-fold serial dilutions ranging from 10^4^ to 10^0^ copies. The RT-qPCR assay used was previously validated to have a C_t_ threshold less than 40 for positive samples.

Samples testing positive for SARS-CoV-2 RNA were cultured using the remaining eluate. The eluate was passed through a 0.45 µL syringe filter and concentrated down to 200 µL using an Amicon Ultra-4 30 kDa Ultracel-PL filter (Millipore Sigma, St. Louis, Missouri, United States), then inoculated onto T25 flasks of Vero E6 cells overexpressing TMPRSS2 and ACE2 (gift from Barney Graham, NIH). Culture media aliquots were removed and placed in Buffer AVL (lysis buffer) (Qiagen, Hilden, Germany) before incubation and at 4 days post infection, when they were also examined for cytopathic effect. Flasks were fixed with 4% paraformaldehyde and stained with a 0.05% crystal violet solution. RNA extraction from AVL buffer aliquots was performed using QIAamp viral RNA Mini kit (Qiagen, Hilden, Germany). The culture medium of the inoculated T25 flasks were tested by RT-qPCR prior to and following the 4-day incubation to determine if there was an increase in RNA copy number (determined by standard curve as described above).

*COVID-19 status of previous occupant of sampled ED rooms*

Medical records were reviewed to determine the COVID-19 status of the room occupant immediately prior to sampled rooms.

*Relationship between patient symptom presentation and ED surface SARS-CoV-2 contamination*

The electronic health record was used to determine days since COVID-19 symptom onset and symptom severity for patients positive during sampling. For all calculations on patient severity and days since symptom onset, only patients with confirmed COVID-19 were included in the analysis. The relationship of room contamination according to COVID-19 severity or time since symptom onset was analyzed with the independent samples median test.

*Statistical analysis*

This study was designed as an exploratory observational design to determine SARS-CoV-2 surface contamination in various clinical settings. As this was performed in a pandemic setting with wide fluctuations in community incidence, this study was not powered to compare surface contamination by procedure type. Two hundred rooms were chosen to sample through estimation of resources allocated to the research team. IBM SPSS version 28.0 was used for statistical analysis with a two-sided p-value of < 0.05 deemed significant.

*References*

1. U.S. Centers for Disease Control and Prevention. Research Use Only 2019-Novel Coronavirus (2019-nCoV) Real-time RT-PCR Primers and Probes. Available at: https://www.cdc.gov/coronavirus/2019-ncov/lab/rt-pcr-panel-primer-probes.html. Accessed November 10, 2022.

Supplemental Table 1. Estimated RNA copies per 100 cm^2^ of swabbed surface area for each positive sample.

| **Surface** | **Patient COVID result** | **Patient AGP* status** | **RNA copies per 100 cm^2^** | **RT-qPCR**  **C_t_ value**** |
| --- | --- | --- | --- | --- |
| Vent | Positive | **No AGP** | 686.6 | 33.91 |
| Bedrail | Positive ‡ | **No AGP** | 538.1 | 33.54 |
| Monitor | Positive | **No AGP** | 504.7 | 33.07 |
| Vent | Positive | **No AGP** | 163.6 | 35.86 |
| Monitor | Positive | **AGP** | 147.9 | 34.76 |
| Bedrail † | Positive | **No AGP** | 114.5 | 35.67 |
| Vent | Positive | **No AGP** | 77.8 | 37.47 |
| Bedrail | Positive | **AGP** | 73.9 | 37.1 |
| Vent | Positive | **No AGP** | 71.7 | 37.59 |
| Equipment | Positive | **AGP** | 64.8 | 36.85 |
| Bedrail | Positive | **AGP** | 59.0 | 38.34 |
| Vent | Positive | **No AGP** | 50.8 | 36.54 |
| Procedure light | Negative | **AGP** | 50.1 | 36.00 |
| Equipment | Negative | **AGP** | 46.8 | 36.65 |
| Door | Negative | **AGP** | 39.4 | 39.1 |
| Monitor | Positive | **AGP** | 37.2 | 38.49 |
| Vent | Positive | **No AGP** | 36.6 | 36.99 |
| Door | Positive | **No AGP** | 36.1 | 38.21 |
| Door | Negative | **AGP** | 32.3 | 37.63 |
| Vent | Positive | **No AGP** | 28.6 | 37.33 |
| Equipment | Negative | **AGP** | 25.8 | 39.28 |
| Vent | Positive | **AGP** | 22.3 | 39.48 |
| Monitor | Negative | **AGP** | 22.2 | 39.19 |
| Bedrail | Negative | **AGP** | 18.2 | 39.2 |
| Equipment | Negative | **AGP** | 15.9 | 39.65 |
| Vent | Positive | **No AGP** | 14.9 | 38.23 |
| Bedrail | Positive | **No AGP** | 13.5 | 38.61 |
| Bedrail | Positive | **No AGP** | 12.8 | 38.68 |
| Procedure light | Negative | **AGP** | 11.9 | 39.78 |
| Vent | Negative | **AGP** | 9.6 | 39.26 |
| Vent | Negative | **AGP** | 9.6 | 38.12 |
| Vent | Negative | **AGP** | 7.5 | 38.49 |
| Bedrail | Positive | **AGP** | 7.4 | 39.45 |
| Bedrail | Positive | **No AGP** | 6.4 | 39.64 |
| Vent | Positive | **No AGP** | 5.0 | 39.72 |
| Bedrail | Positive | **AGP** | 4.7 | 39.86 |
|  |  |  |  |  |

* AGP: Aerosol-generating procedure; **RT-qPCR C_t_ value is value for whole sample and not divided by sample surface area; † Sample from which viable SARS-CoV-2 virus was recovered. ‡ COVID-19-positive by self-report. Not tested at Yale New Haven Hospital.

Supplementary Figure 1A. Median number of days from symptom onset to emergency department presentation in rooms with or without detectable SARS-CoV-2 contamination.
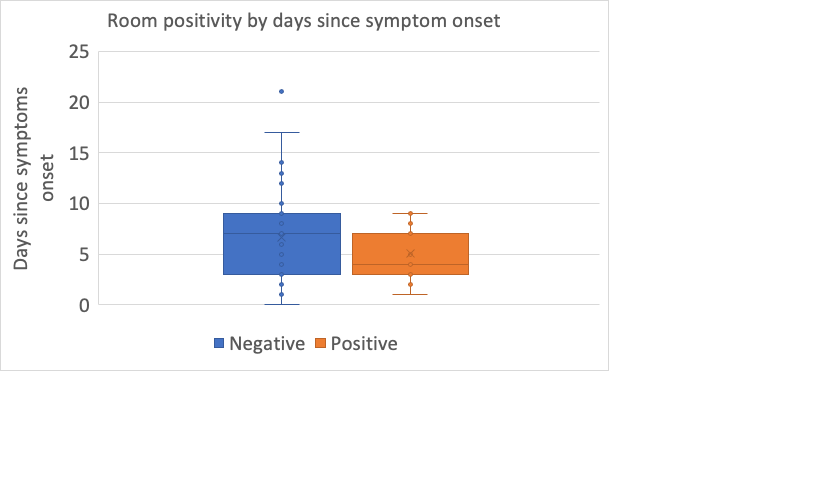


Supplementary Figure 1B. Median severity of patient illness on arrival in rooms with or without detectable SARS-CoV-2 environmental contamination.


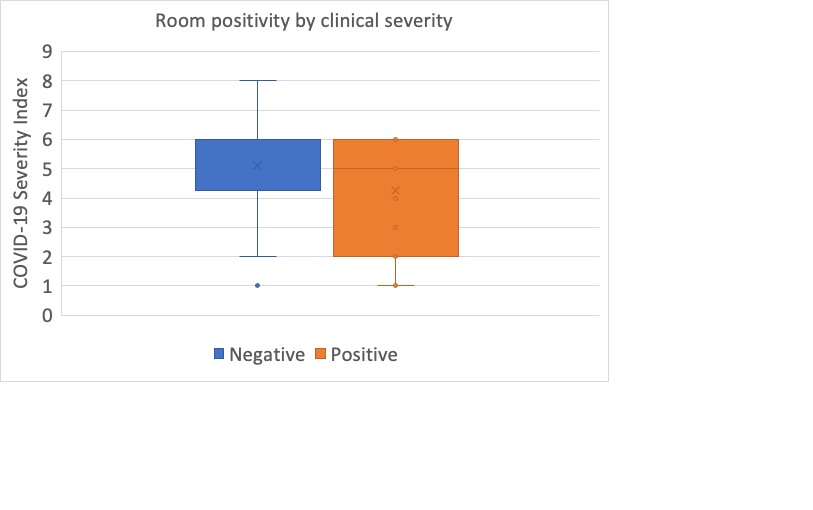

Supplement: Supplementary file 1 [file S0899823X23001836sup001.docx]
